# Potentially handicapped but otherwise functional: Malformations in prey capture tools show no impacts on octopus life

**DOI:** 10.1002/ece3.6903

**Published:** 2020-10-17

**Authors:** Fernando Ángel Fernández‐Álvarez, Marc Farré, Antoni Sánchez‐Márquez, Roger Villanueva, Oscar Escolar, Joan Navarro

**Affiliations:** ^1^ Institut de Ciències del Mar (CSIC) Barcelona Spain; ^2^ Ryan Institute and School of Natural Sciences National University of Ireland Galway Galway Ireland

**Keywords:** geometric morphometrics, Larval ecology, morphology, planktonic ecology, teratology, trophic ecology

## Abstract

Larval mortality is a keystone ecological factor for many benthic octopus since it mostly occurs before their settlement in the sea bottom as benthic juveniles. The literature had revealed that records of adult animals with morphological abnormalities (teratologies) are fewer in species with complex life cycle than in those with direct development. This is a direct consequence of the morphological, physiological, and development challenges that the transition from the larval to the adult morphology represents. During a routine fishing sample, we found an immature female horned octopus with additional buccal structures in two suckers of its ventral arms, likely rendering these suckers as inefficient. Based on the literature about the natural history of octopus, we provide evidence that these abnormalities were present at the moment of hatch. We evaluated the impact of the teratologies by comparing the shape of the buccal beaks and the trophic niche of the individual with five normal conspecifics. Although the beaks showed a different shape than normal individuals, the trophic niche was similar. Surprisingly, the teratological condition of the individual likely had no severe impacts on its life, even though it likely represents a handicap for its survival during its planktonic life. We also comment on other previous records from the literature of teratological adult octopus to highlight the amazing adaptive capacity of octopus to deal with challenging morphologies.

## Graphical abstract

An immature female horned octopus with additional buccal structures in two suckers of its ventral arms, rendering these suckers as inefficient. Although the beaks showed a different shape than normal individuals, the trophic niche was similar. Thus, the teratological condition of the individual likely had no severe impacts on its life, even though it likely represents a handicap for its survival during its planktonic life.

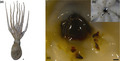

## 1 INTRODUCTION

The population mortality of marine fauna with complex life cycles is usually concentrated in the planktonic stages. This is mainly due to the ecological, physiological, morphological, and developmental challenges that entail the transition from the larval form to the adult morphology (Vaughn & Alen, 2010). A clear example is many benthic octopuses, where the higher natural mortality is concentrated in the early phase of their life cycle (paralarval stage). Because these marine predators have a short lifespan (usually around one year) and die after a single reproductive event, early survival is crucial for the population dynamics of octopus species from the continental shelf. For this reason, their population maintenance mainly depends on the survival of the planktonic paralarval cohorts.

Overall, the survival rate of individuals with teratologies (developmental malformations) is expected to be lower than normal conspecifics. This is especially true in species with complex life cycles. Not surprisingly, teratologies are more frequently reported in the adults of animals with direct development than in those with complex life cycles involving metamorphoses (see Fernández‐Álvarez et al., 2011 and references therein), indicating the lethality of some of those anomalies, especially during early developmental stages.

**Figure 1:**
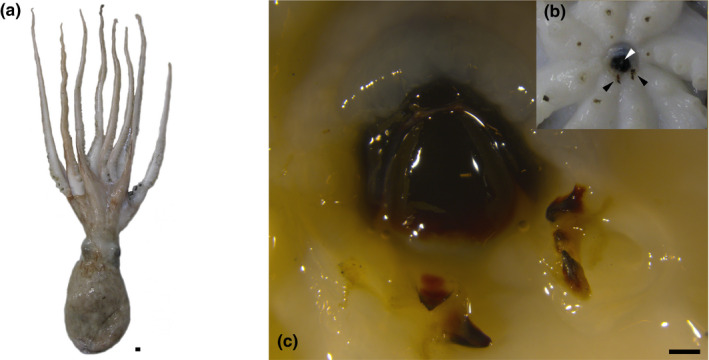
(a) Dorsal view of the teratological horned octopus, *Eledone cirrhosa*, individual, currently stored at the Biological Reference Collections of the Marine Science Institute (CBR‐ICM) under the catalog number ICMC000317. (b) Oral view of the arm crown showing the normal beaks (white arrowhead) and the teratological suckers with additional beaks (black arrowheads). (c) Close‐up of the buccal area. Scale bars: 1 mm

**Figure 2:**
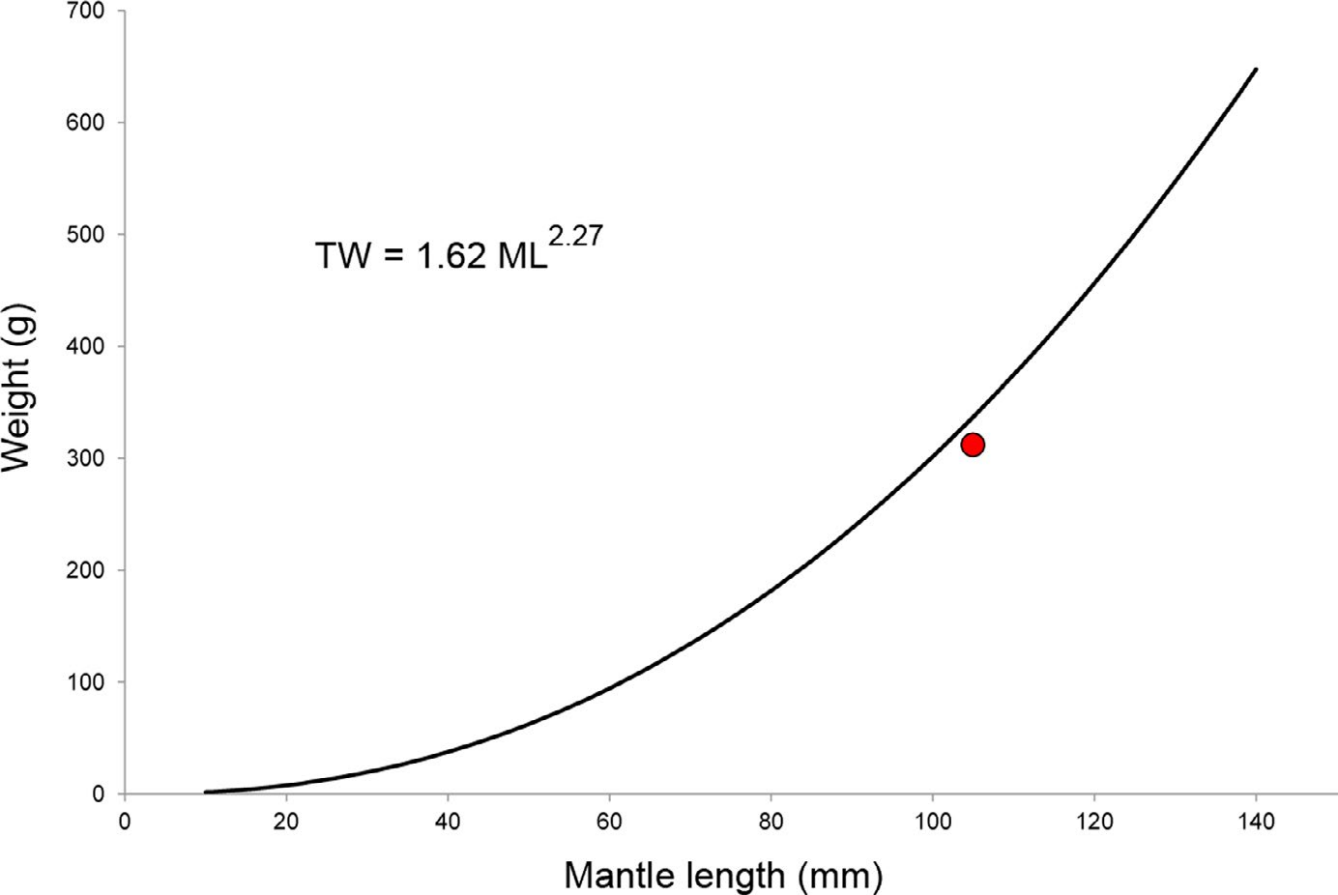
Graph showing the length/weight relationship for horned octopus based on the equation provided by Jereb et al. (2015); the red point signals the actual weight and length of the teratological individual. Abbreviations: TW, total weight; ML, mantle length

During a routine fish sampling using bottom trawling, we found a single‐horned octopus (*Eledone cirrhosa*) displaying additional buccal pieces at the level of the first sucker of both ventral arms (see Figure 1). The individual was collected together with other conspecifics at 149 m depth on November 28 2018 off Roses (Catalonia, Spain), northwestern Mediterranean Sea. This teratological individual was a subadult female with a 105 mm dorsal mantle length and 312 g total body mass and showing an apparently normal external (Figure 1a) and internal morphology, including gonads resembling those of normal immature females. Moreover, it fit the length/weight relationship published for this species in the northwestern Mediterranean (Jereb et al., 2015): The expected weight of a 105 mm ML individual should be around 336 g, close to the actual weight of the teratological specimen. This suggests that its condition was similar to normal individuals from the same population (Figure 2). In this individual, additional small beaks were present in the first suckers of the ventral pair of arms (Figure 2). The morphology of the additional beaks resembled that of the normal chitinous beaks of the individual, including a darkly pigmented rostrum, rudimentary wings, and lateral walls (Figure 1b‐c), although its shape was narrower and irregular (Figure 1c). They measured approximately 1.5 mm in rostral length (see Clarke, 1986 for a formal description of the beaks). The additional beaks occupied approximately 1/3 of the surface area of each sucker, rupturing its muscular assemblage. The right ventral arm contained two structures resembling upper beak morphology, with the inner part directed toward the dorsal surface of the animals as in normal upper beaks happen. In the left ventral arm, there were three structures: The one closer to the mouth resembled the lower beak with irregular wings, the one placed distally resembled a very narrow upper beak, while the intermediate structure was comparatively smaller, poorly developed, and difficult to allocate to any known cephalopod beak shape but with an unmistakable developing rostrum.

Horned octopus paralarvae hatch with 4.5 mm mantle length (ML) and eight small arms equipped with eight suckers each (Mangold et al., 1971). Since hatchling suckers are not replaced through its life, the first eight suckers on each arm are the very same the individual used for prey capture and handling during their first days of its planktonic life. The beaks and the arm suckers in octopus are formed during the last third of the embryonic development (Armelloni et al., 2020); therefore, the additional beaks of this teratological individual must have been developed during its embryonic life and accompanied the octopus through its life.

**Figure 3:**
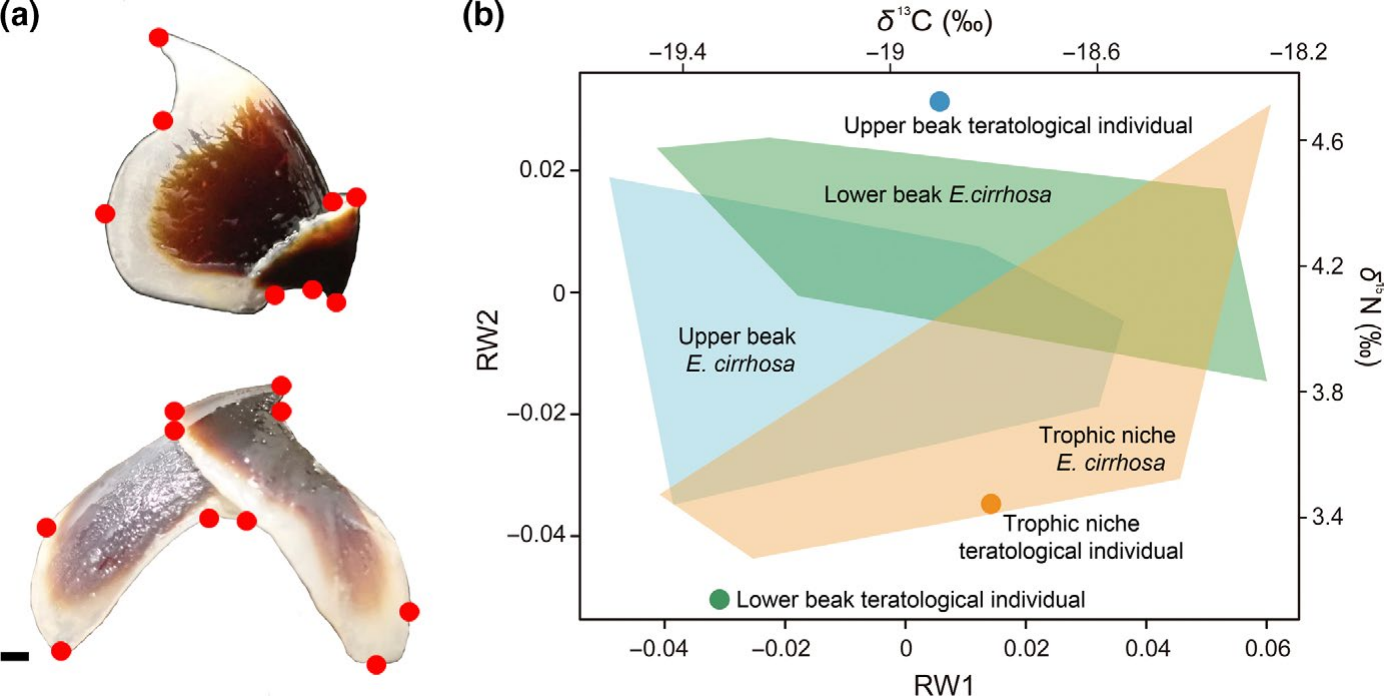
(a) Detail of the landmarks defined in this study for the upper (up) and lower (down) beaks. (b) Morphospaces of the upper (in shaded blue) and lower (in shaded green) beak shape and isotopic space (in shaded orange) of the analyzed horned octopus individuals, highlighting the position (colored points) of the teratological specimen in each space. Scale bar: 1 mm

In addition to the teratological suckers, we could also detect a degree of difference in the buccal beak morphology of this individual by using geometric morphometric (GM) methods. Specifically, we compared the shape of the upper and lower buccal beaks between this individual and five conspecifics, collected from the same area, of similar size, sex, and sexual maturity stage. The use of GM allowed us to accurately describe the morphological shape of the lower and upper beak of each individual examined (Figure 3a; for more details of shape description, see Neige and Dommergues (2002); and Adams et al. (2004) for a detailed explanation of the method). GM analysis showed that the shape of both upper and lower beaks of the teratological individual differed from those of the five normal analyzed conspecifics, occupying an extreme and isolated position within both morphospaces (Figure 3b).

Given that the arms and the buccal mass have primary functions in the prey capture, handling, and feeding process of octopus, these teratologies could place constraints associated with its feeding behavior, perhaps limiting the consumption of similar prey compared to conspecifics without any teratological trait and, thus, negatively affecting survival. Assuming this, the presence of these teratologies in a subadult octopus led us ask an ecological question: How is possible that a teratological individual survived so long? To answer this, we compared the trophic niche of the teratological individual with those of the same five conspecifics without any teratology using stable isotope analyses of nitrogen (*δ* ^15^N) and carbon (*δ* ^13^C) on their beaks. These trophic markers are useful to determine the trophic niche occupied by cephalopods (Navarro et al., 2013) because *δ* ^15^N informs trophic level of the consumer while *δ* ^13^C values are useful indicators of the dietary source of carbon. Unexpectedly, stable isotope values revealed that the teratological individual and the conspecifics did not differ in their trophic niche (Figure 3b). Thus, despite of the malformations in the suckers and the differences in the buccal beak shape, the trophic markers showed that the teratological individual is likely to have fed on similar feeding resources to the conspecifics.

Octopus paralarvae feed on planktonic prey, which they capture with the aid of the suckers and arms, just before using their beaks to both wound the prey and inoculate its body with digestive enzymes and finally to suck‐up the predigested content (Villanueva & Norman, 2008). The internal pressure reduction that enables an octopus sucker to function requires the strong action of its muscles coupled with an efficient seal against the surface of the prey (Kier & Smith, 2002), helped by the mucus film that covers the sucker surface in octopus paralarvae (Accogli et al., 2017). Thus, the presence of the extranumeral beaks in the suckers could not only compromise the tridimensional structure of the musculature, but it also could disable the sealing of the sucker against the prey surface, hindering prey capture, holding, and subduing and, consequently, rendering the affected suckers inefficient. Due to their position near the mouth, the basal suckers from each arm are paramount during feeding operations, since they keep the prey in the right position for killing and eating it. In addition, suckers have mechano‐ and chemoreceptors used to select the food quality ingested (Villanueva & Norman, 2008). Therefore, it seems logical to hypothesize that two malfunctional suckers in this position represent an important disadvantage in comparison with conspecifics, particularly during the delicate paralarval period.

Previous literature shows a few examples of teratological octopus which were able to reach adulthood even with characters that compromise their survival, such as the absence of a gill or with the arms heavily branched (see Torres et al., 2018 and references therein). Toll and Binger (1991) found two fully mature male individuals of different octopus species with six and ten arms. This illustrates that octopus show an adaptive ability and resilience against malformed bodies, likely higher than other animals with similarly complex ontogeny, and how impressive is their ability to survive teratologies.

The developmental evidence supports that this teratological horned octopus would have had to adapt to this malformation since the moment it has born. Octopus species are renowned for their behavioral plasticity to deal with changes in their environment (Boyle & Rodhouse, 2005), so maybe the resilience of this individual involved changes in the use of other unaffected suckers. The results from this communication suggest that this plasticity may mitigate against the high mortality normally associated with planktonic life, where even a malformed planktonic octopus is capable of adjusting its behavior to prosper and cheat death under higher selective forces, such as those paralarvae should face during their life in the water column.

## CONFLICT OF INTERESTS

There is no conflict of interest.

## AUTHOR CONTRIBUTION

**Fernando Ángel Fernández‐Álvarez:** Conceptualization (lead); Investigation (lead); Supervision (lead); Writing‐original draft (lead). **Marc Farré:** Formal analysis (supporting); Investigation (equal); Writing‐review & editing (supporting). **Antoni Sánchez‐Márquez:** Data curation (lead); Formal analysis (lead). **Roger Villanueva:** Investigation (equal); Writing‐review & editing (supporting). **Oscar Escolar‐Sánchez:** Data curation (supporting); Investigation (equal). **Joan Navarro:** Conceptualization (equal); Formal analysis (supporting); Investigation (equal); Resources (lead); Writing‐review & editing (supporting).

## Supporting information

Appendix S1

## Data Availability

All relevant data are included in the manuscript. Raw data are provided in the Appendix S1 and in the Spanish National Research Council (CSIC) virtual repository under the doi accession number 10.20350/digitalCSIC/12622.
