# Supplementary material for: Potentially handicapped but otherwise functional: Malformations in prey capture tools show no impacts on octopus life
Source: Ecol Evol. 2020 Oct 17;10(23):12685–9. doi: 10.1002/ece3.6903 (PMC7713964; doi:10.1002/ece3.6903)
Supplement: Supplementary file 1 — Appendix S1 [file ECE3-10-12685-s001.docx]

APPENDIX TO “**POTENTIALLY HANDICAPPED BUT OTHERWISE FUNCTIONAL: MALFORMATIONS IN PREY CAPTURE TOOLS SHOW NO IMPACTS ON OCTOPUS LIFE**”

by Fernando Ángel Fernández-Álvarez^1,2^, Marc Farré^1^, Antoni Sánchez-Márquez^1^, Roger Villanueva^1^, Oscar Escolar^1^, Joan Navarro^1^

^1^Institut de Ciències del Mar (CSIC), Passeig Marítim 37-49, E-08003 Barcelona, Spain.

^2^Ryan Institute and School of Natural Sciences, National University of Ireland Galway, Galway, Ireland, H91 TK33.

**Correspondence**: Fernando Ángel Fernández-Álvarez. Ryan Institute and School of Natural Sciences, National University of Ireland Galway, Galway, Ireland, H91 TK33. E-mail: f.a.fernandez.alvarez@gmail.com

**ORCID codes**: FÁF-Á: 0000-0002-8679-7377, MF: 0000-0002-2263-4800, AS-M: 0000-0003-0414-4928, RV: 0000-0001-8122-3449, OE: 0000-0002-1785-0138, JN: 0000-0002-5756-9543.

**Table S1.** Collecting date, weight (g), mantle length (ML, mm), sex and maturity stage of the studied horned octopus specimens.

| **Sample ID** | **Date** | **Weight** | **ML** | **Sex** | **Maturity stage** | **Comments** |
| --- | --- | --- | --- | --- | --- | --- |
| Ecirr_01 | 28/11/2018 | 258 | 107 | Female | Immature | Normal morphology |
| Ecirr_03 | 28/11/2018 | 368 | 122 | Female | Immature | Normal morphology |
| Ecirr_05 | 28/11/2018 | 312 | 105 | Female | Immature | **Teratological morphology** |
| Ecirr_10 | 28/11/2018 | 255 | 100 | Female | Immature | Normal morphology |
| Ecirr_12 | 28/11/2018 | 235 | 105 | Female | Immature | Normal morphology |
| Ecirr_15 | 28/11/2018 | 204 | 88 | Female | Immature | Normal morphology |

**Table S2.** Morphometric data (relative warps, RWs) of the upper beak for the studied horned octopus specimens.

| **Sample ID** | **RW1** | **RW2** | **RW3** | **RW4** | **RW5** |
| --- | --- | --- | --- | --- | --- |
| Ecirr_01 | 3.19E-02 | -1.87E-02 | 2.23E-02 | -8.22E-03 | 5.63E-03 |
| Ecirr_03 | 3.61E-02 | -4.60E-03 | -1.38E-02 | -7.56E-03 | -1.06E-02 |
| Ecirr_05 | 8.19E-03 | 3.14E-02 | 1.33E-02 | 1.68E-02 | -2.99E-03 |
| Ecirr_10 | -3.88E-02 | -3.48E-02 | -1.76E-03 | 1.22E-02 | -2.19E-03 |
| Ecirr_12 | -4.94E-02 | 1.90E-02 | 2.45E-03 | -1.67E-02 | -3.19E-04 |
| Ecirr_15 | 1.20E-02 | 7.64E-03 | -2.25E-02 | 3.60E-03 | 1.05E-02 |

**Table S3**. Morphometric data (relative warps, RWs) of the lower beak for the studied horned octopus specimens.

| **Sample ID** | **RW1** | **RW2** | **RW3** | **RW4** | **RW5** |
| --- | --- | --- | --- | --- | --- |
| Ecirr_01 | -2.29E-02 | 2.54E-02 | -1.28E-02 | 1.13E-02 | 7.45E-03 |
| Ecirr_03 | -1.81E-02 | -6.14E-04 | 4.84E-02 | -1.38E-03 | 5.88E-04 |
| Ecirr_05 | -3.03E-02 | -5.07E-02 | -1.27E-02 | 6.18E-03 | -1.94E-03 |
| Ecirr_10 | -4.16E-02 | 2.37E-02 | -1.54E-02 | -1.44E-02 | -4.36E-03 |
| Ecirr_12 | 5.30E-02 | 1.69E-02 | -1.07E-03 | 1.09E-02 | -6.65E-03 |
| Ecirr_15 | 5.98E-02 | -1.46E-02 | -6.43E-03 | -1.26E-02 | 4.91E-03 |

**Table S4**. Isotopic data from the beaks of the studied horned octopus specimens.

| **Sample ID** | **Delta 13C x 1000** | **Delta 15N x 1000** |
| --- | --- | --- |
| Ecirr_01 | -19.26 | 3.27 |
| Ecirr_03 | -18.26 | 4.7 |
| Ecirr_05 | -18.8 | 3.43 |
| Ecirr_10 | -19.44 | 3.46 |
| Ecirr_12 | -18.95 | 3.77 |
| Ecirr_15 | -18.44 | 3.51 |
